# Urachal Cyst Diagnosed by Point-of-care Ultrasound

**DOI:** 10.5811/cpcem.2017.9.34905

**Published:** 2017-10-18

**Authors:** Vigil James, Jade Seguin, Charisse W. Kwan

**Affiliations:** * University of Toronto, The Hospital for Sick Children, Department of Pediatrics, Division of Emergency Medicine, Toronto, Canada; † McGill University, The Montreal Children’s Hospital, Department of Pediatrics, Division of Emergency Medicine

## Abstract

Irreducible umbilical swelling in infants is considered a surgical emergency because a delay in surgical intervention for an incarcerated umbilical hernia can lead to bowel ischemia and necrosis. We report two patients who presented to a pediatric emergency department with history and symptoms of irreducible umbilical mass suggestive of umbilical hernia. Point-of-care ultrasound was used at the bedside to demonstrate the presence of urachal cyst remnants and accurately guided the care of these children.

## INTRODUCTION

Irreducible umbilical swelling in infants is considered a surgical emergency because a delay in surgical intervention for an incarcerated umbilical hernia can lead to bowel ischemia and necrosis.^1^ Infants can be stable or unstable and present with or without associated features of vomiting, irritability, and abdominal distension, which may guide investigations and management. Although the diagnosis of umbilical hernia incarceration is high on the differential diagnosis, it is rare and other conditions need to be considered, including urachal cyst, midline dermoid cyst, hemangiomas, umbilical polyps and omphalomesenteric remnants. It often requires timely radiologic investigations and urgent consultation with the surgical team for definitive management. We report the first cases of two patients, one of whom was referred to us as an incarcerated umbilical hernia and the other with an infected irreducible umbilical mass. Point-of-care ultrasound (POCUS) performed by emergency providers helped diagnose urachal cyst as the cause for these umbilical masses.

## CASE PRESENTATION

### One

A previously healthy 10-month-old female presented to our emergency department (ED) with a two-day history of multiple episodes of non-bilious vomiting and abdominal distension. One week prior to presentation, the parents had noticed an irreducible umbilical mass. There was no history of diarrhea or fever. On examination she was alert, her heart rate was 108 beats per minute, blood pressure was 84/50 millimeters of mercury (mmHg), capillary refill was less than two seconds, and there were no signs of dehydration. Her abdomen was distended but soft and bowel sounds were present. An irreducible tense umbilical mass of 3×3 centimeters (cm) was noted. The skin was normal around the umbilicus, but appeared shiny over the mass. Her cardiovascular, respiratory and central nervous system examination was unremarkable.

**Image 1:**
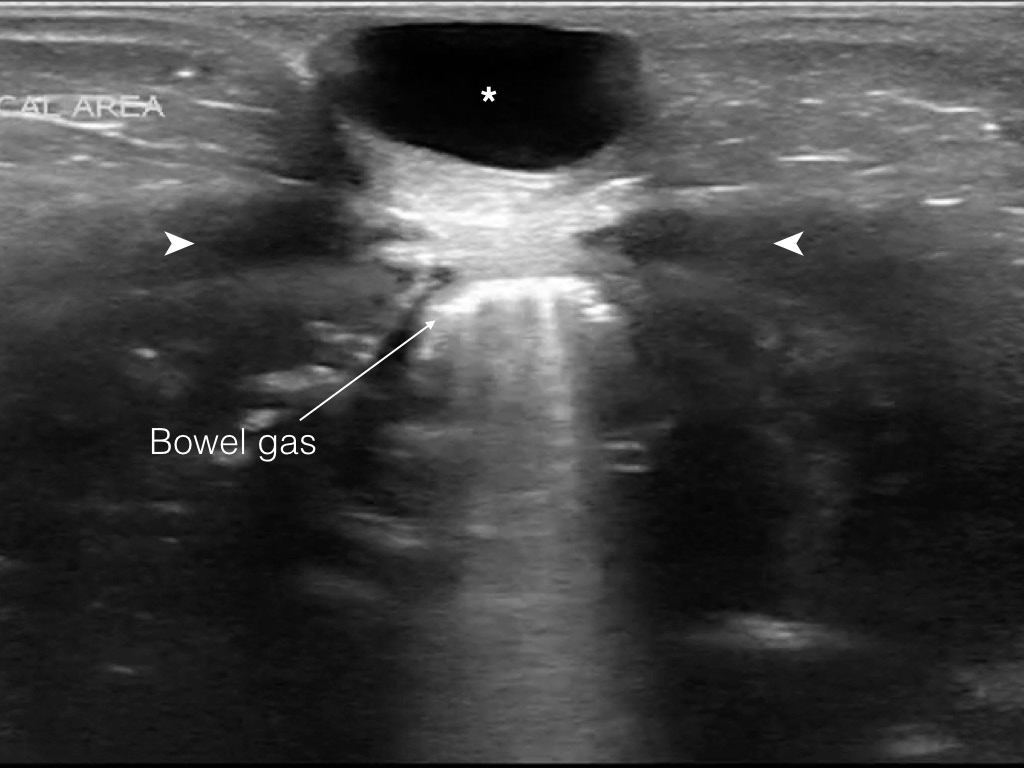
A linear transducer (14-5MHz) image in transverse view demonstrating urachal cyst (*) located between the abdominal recti (†) muscles, superficial to the peritoneal cavity. The hyperechoic lines with posterior shadowing represent bowel gas.

An initial diagnosis of incarcerated umbilical hernia was considered. The pediatric emergency medicine (PEM) fellow performed a POCUS using linear transducer (14-5MHz), which showed a well-circumscribed hypoechoic structure suggestive of a fluid-filled sac. The sac did not communicate with the intra-abdominal cavity, and there was no evidence of bowel loops or peritoneum in the sac (Image 1). A diagnosis of urachal cyst was considered. In view of the multiple episodes of non-bilious vomiting, a trial of oral rehydration therapy was done in the ED for two hours, which the baby tolerated well. On reassessment, the abdomen was soft and there were no signs of dehydration. The baby was discharged home with a diagnosis of acute gastroenteritis with no dehydration and an incidental finding of probable urachal cyst. A comprehensive ultrasound done by the radiologist the next day confirmed the presence of urachal cyst with no evidence of vesico-urachal diverticulum, and the baby was followed up in the outpatient urology clinic.

### Two

A two-year-old male presented to the ED with redness and a prominent protrusion in the umbilical area for two days. There was no fever, vomiting, abdominal pain or discharge from the umbilicus. He had a recent hospitalization for urinary tract infection requiring intravenous antibiotics. On examination he was alert, his heart rate was 96 beats per minute, blood pressure was 104/72 millimeters of mercury (mmHg) and capillary refill was less than three seconds. The abdomen was soft and non-tender, with no guarding or rebound tenderness. An umbilical mass of 2×2 cm was noted, and the overlying skin was erythematous and tender. The skin around the mass was not erythematous or indurated. The genitourinary examination showed normal, bilaterally descended testes and no inguinal hernia.

**Image 2:**
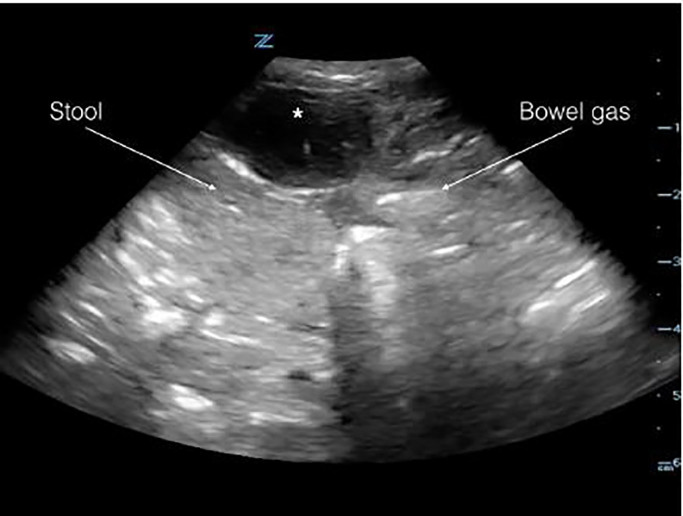
A linear transducer (14-5MHz) image in transverse view demonstrating urachal cyst (*) located superficial to the peritoneal cavity. The hyperechoic lines with posterior shadowing represent bowel gas and heterogeneous material inside the peritoneum within the bowel loops represent stool.

The PEM fellow performed a POCUS using linear transducer (14-5MHz), which revealed a protruding hypoechoic structure suggestive of a fluid-filled sac that did not communicate with the intra-abdominal cavity. There were no bowel loops within the sac and there was no cobblestoning of the overlying skin (Image 2). A probable diagnosis of infected urachal cyst was considered. Urology was consulted and agreed with the diagnosis of infected urachal cyst/remnant. The patient was started on oral cefprozil for 10 days and advised to follow up in the urology clinic. A comprehensive ultrasound done by the radiologist the next day revealed the urachal cyst with a vesico-urachal diverticulum.

**CPC-EM Capsule**

What do we already know about this clinical entity?

*Irreducible umbilical swelling in infants is a surgical emergency because a delay in surgical intervention for an incarcerated umbilical hernia can lead to bowel ischemia and necrosis.*

What makes this presentation of disease reportable?

We present the first report of point-of-care ultrasound being used in the diagnostic algorithm for irreducible umbilical swelling in infants.

What is the major learning point?

Urachal cyst remnant is a rare but important differential diagnosis for irreducible umbilical swelling in infants.

How might this improve emergency medicine practice?

Bedside ultrasound is a simple and useful clinical adjunct that can be used to facilitate a quick and early diagnosis of urachal cyst remnants in infants and children.

## DISCUSSION

Median umbilical ligament, or urachus, is a midline tubular structure that extends upward from the anterior dome of the bladder toward the umbilicus. It is a remnant of two embryonic structures: cloaca, which is the cephalic extension of the urogenital sinus, and allantois, which is derived from yolk sac.^2^ The tubular structure usually disappears before birth. The persistence of the urachal remnant can give rise to a spectrum of clinical conditions such as vesico-urachal diverticulum (distal communication to bladder persists); patent urachus (entire tubular structures remains open); umbilical-urachal sinus (opens proximally into umbilicus); and urachal cyst (both ends of the canal obliterate leaving only central portion open).^2^–^4^ In children, urachal anomalies most commonly present as umbilical mass, umbilical drainage or pain due to infection of the cyst.^5^–^7^

POCUS evaluation of the umbilical mass is ideally done using a high-frequency linear probe to visualize superficial structures; however, a phased array or curvilinear probe can also be used. The umbilical mass must be visualized in both transverse and longitudinal planes. Use of adjuncts such as color and power Doppler can provide vital information about the blood flow into the umbilical area and surrounding structures. The skin around the umbilicus should be scanned to look for cobblestoning. This finding, in conjunction with clinical signs such as skin erythema and tenderness, may suggest cellulitis.

The sonographic appearance of urachal cyst is a fluid-filled anechoic structure, which lies between the skin and anterior abdominal wall in the midline of the abdomen, below the umbilicus. No bowel content or peristalsis is visualized.^8^ If the cyst becomes infected, the contents can appear heterogeneous.^3^ Concurrent scanning of the suprapubic area of the abdomen to assess the bladder with a low-frequency (phased array) probe might demonstrate heterogeneous echogenic mass above the bladder in case of a vesico-urachal diverticulum.

## CONCLUSION

We present the first reports of point-of-care ultrasound application by a pediatric emergency physician to identify urachal remnants in children. Ultrasonography can readily identify urachal cyst greater than a few millimeters in size.^3^–^6^ By integrating POCUS into clinical examination of children presenting with umbilical mass, the treating physicians were able to make a rapid bedside diagnosis of urachal cysts. This could help avoid unnecessary attempts and/or manipulations for reduction of the umbilical mass, the associated sedation required and ED surgical consultation for an incarcerated umbilical hernia. POCUS in the ED is known to result in increased time efficiency in emergency patient care.^9^,^10^ Better understanding of the role of POCUS in the diagnosis of this congenital anomaly can help to narrow differential diagnosis, expedite the diagnosis and management of these children, avoid unnecessary and potentially harmful interventions and tailor downstream care. When used carefully with full understanding of limitations, it can also help to reduce physician cognitive burden and avoid cognitive bias. These cases cannot be used to make conclusions about the diagnostic accuracy of POCUS in this condition but warrants further research into this promising modality.
